# Long-term outcome of patients with Ménière’s disease following cochlear implantation: a comprehensive outcome study with validated assessment tools

**DOI:** 10.1007/s00405-024-08690-1

**Published:** 2024-05-20

**Authors:** Miray-Su Yılmaz Topçuoğlu, Peter K. Plinkert, Mark Praetorius, Sara Euteneuer

**Affiliations:** 1 Department of Otorhinolaryngology, University Hospital Heidelberg, Im Neuenheimer Feld 400, 69120 Heidelberg, Germany; 2 Department of Otorhinolaryngology, University Hospital Hamburg-Eppendorf, Hamburg, Germany

**Keywords:** Cochlear implantation, Hearing outcomes, Ménière’s disease, Quality of life, Tinnitus, Vertigo

## Abstract

**Purpose:**

Patients suffering from Ménière’s disease (MD) experience vertigo, and impairments in hearing and quality of life (QoL). This study aims to investigate the impact of cochlear implantation (CI) on various aspects affecting patients with MD.

**Methods:**

A single tertiary centre’s CI database for CI recipients with MD between 2014 and 2022 was screened retrospectively. Hearing, vertigo, tinnitus symptoms, and hearing-related QoL were assessed. Pre- and postoperative hearing tests in conjunction with subjective outcome measures by visual analogue scale (VAS) and validated tools such as the Dizziness Handicap Inventory (DHI), Tinnitus Handicap Inventory (THI) and Nijmegen Cochlear Implant Questionnaire (NCIQ), as well as the assessment of the pre- and postoperative Functional Level Scale (FLS) were examined.

**Results:**

Eleven ears were included (median age: 59 years at implantation). Following implantation, there was a significant enhancement in Word Recognition Scores at sound levels of 65 dB and 80 dB compared to before treatment (preop vs. 12 months postop: p = 0.012). However, no significant enhancement was observed for 50 dB. MD-related impairments improved significantly postoperatively, as measured by the VAS (vertigo: p = 0.017; tinnitus: p = 0.042), DHI (p = 0.043), THI (p = 0.043) and NCIQ (p < 0.001). The FLS improved significantly (p = 0.020).

**Conclusion:**

CI has positive effects on all areas examined in our cohort. However, discrimination of speech at low sound pressure levels remained problematic postoperatively. In patients suffering from MD, the prioritized treatment goals include not only improved hearing but also the rehabilitation of vertigo and tinnitus, as well as the enhancement of QoL. Validated instruments are useful screening tools.

## Introduction

Ménière’s disease (MD) is a disorder affecting the inner ear that is likely caused by a hydrops of the endolymphatic fluid [1–3]. Common symptoms include sudden vertigo, tinnitus, a feeling of fullness in the affected ear, and initially fluctuating hearing loss that later might progress to deafness [1, 2, 4–6]. The prevalence is estimated to be around 20–200 cases per 100.000 people [1, 2, 4, 7–9] with a higher incidence among women and peaks between the age of 40 and 60 [4, 7–9]. Both ears are affected in around 30% of cases over a lifetime [4, 6, 10]. Quality of life (QoL) is reduced in patients with MD who suffer from both physical and psychological impairments [1, 11]. Treatment options range from oral betahistine or corticosteroids to the intratympanic application of corticosteroids, as well as endolymphatic sac decompression [1]. Chemical and surgical ablative treatments, such as intratympanic gentamicin application, surgical labyrinthectomy or neurectomy are also treatment methods [1].

Cochlear implantation (CI) has been found to enhance the QoL and speech perception in highly hearing-impaired patients [12, 13]. For individuals with MD and progressive hearing loss or deafness that is untreatable with conventional hearing aids, CI can provide significant benefits [6, 14–20]. Those MD patients who undergo CI experience similar benefits to those patients with CI without MD [14, 16, 21–23], and MD patients often report an improvement in hearing and speech perception after undergoing a CI [14–17, 22–24]. Furthermore, they report an improvement in other aspects of QoL, such as relief from vertigo and tinnitus [14–17, 22–24], even though it has been shown, that CI can lead to a postoperative exacerbation of MD in objective tests [25]. However, the full impact of CI on tinnitus and vertigo in MD patients is yet to be fully understood [15, 22].

Due to the complex nature of the disease, it is important to take a comprehensive approach to assessing the condition of patients with MD, rather than focusing on only isolated aspects of their condition. Pure tone audiometry and speech perception [6, 14, 16–18, 21–23, 26, 27] should be complemented by the evaluation of QoL and functional level scale (FLS) [15–17, 21, 23, 26], as well as the validated assessment of vertigo [23, 26, 28] and tinnitus [16, 17, 23, 26]. Several studies have investigated additional outcome aspects of MD patients after CI, in addition to evaluating hearing outcomes [6, 14–17, 21, 26, 28]. But none of these studies took a comprehensive approach to evaluating all important outcome aspects in a single cohort, including the outcome of hearing, vertigo and tinnitus, as well as the hearing-related QoL. Instead, only certain singular outcome aspects were analysed. The objective of this study was to systematically examine a cohort of MD patients who received CI in our department. Our approach used validated tools to assess various outcome aspects and aims to achieve a holistic understanding of the complex disease pattern of MD. To the best of our knowledge, this is the first study to examine this broad range of outcome aspects in one cohort of MD patients with CI using validated tools.

## Materials & methods

### Inclusion criteria & study design

This was a retrospective study conducted at a single tertiary centre. The study was non-randomised, non-controlled, and non-blinded.

**Fig. 1:**
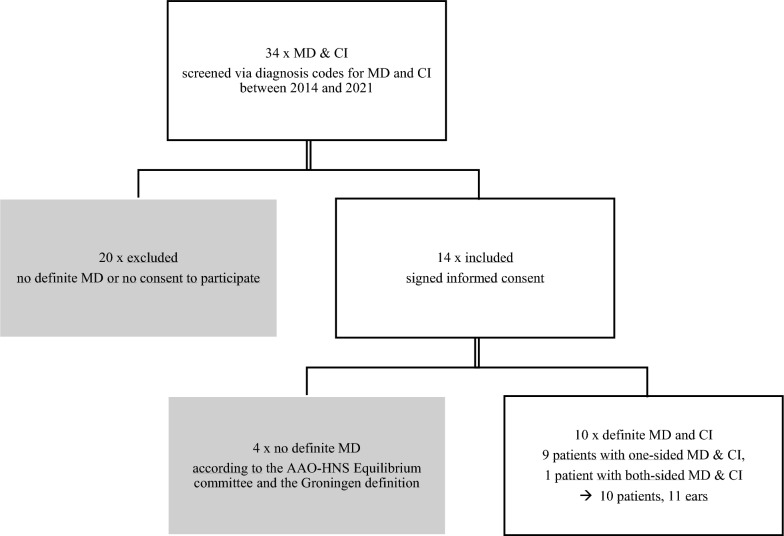
Flowchart of the enrolment process. Of the 34 patients screened for Ménière’s disease (MD) and cochlear implantation (CI) via the diagnosis code, only 14 met the inclusion criteria and provided informed consent. Four patients were later excluded during the second check-up due to not meeting the American Academy of Otolaryngology-Head and Neck Surgery (AAO-HNS) Equilibrium Committee and Groningen definition for definitive MD. Ten patients remained who met the inclusion criteria, including one patient with bilateral MD who underwent two-staged bilateral CI in the investigation period

We included patients diagnosed with definitive Ménière’s Disease as per American Academy of Otolaryngology-Head and Neck Surgery (AAO-HNS) Equilibrium Committee criteria and the Ménière Groningen Definition [1, 5, 27] who underwent CI on the affected ear between 2014 and 2022 in our department. We identified eligible patients through diagnosis codes in the hospital’s digital medical record system. To assess long-term outcomes, only patients with an implantation of at least twelve months ago prior to study participation, and ongoing postoperative follow-up visits in our department were included. Patients who met the aforementioned MD criteria were called and asked whether they would participate in the study. If they consented, they were sent the study documents and the informed consent form by post, due to the challenges of personal contact at the hospital during the recruitment phase in times of the corona pandemic. In total, 10 patients and 11 ears with definite MD and CI were enrolled. The enrolment process is outlined in Fig. 1.

### Ethics

The study was granted approval by the local ethics committee (S-319/2021). All ethical standards as per the current revision of the Declaration of Helsinki of 2013 were adhered to. Participation in the study was strictly limited to individuals over 18 years of age who provided written informed consent.

### Assessed data

Patient data including hearing outcome and history, age at implantation, side of implantation, history of previous MD therapies, and the manufacturer of CI were extracted retrospectively from the digital medical records. Further data were extracted from patient-completed questionnaires as their personal experience with their CI, but also including the validated Dizziness Handicap Inventory (DHI), Tinnitus Handicap Inventory (THI), Nijmegen Cochlear Implant Questionnaire (NCIQ), and the FLS. Additionally, patients were asked to evaluate their vertigo- and tinnitus-related impairments using a visual analogue scale (VAS) to compare these results with the results of the validated tools DHI and THI. Patients were asked to complete the DHI, THI and NCIQ and the FLS and VAS twice. Once, they were asked to evaluate the situation before the cochlear implantation from a retrospective perspective and once, they were asked to evaluate the current situation with the cochlear implant. Additionally, patients were asked about their individual most disabling subjective MD symptoms, with the possibility of choosing more than one answer: hearing loss, vertigo, tinnitus, nausea during attacks, days off to work and social life.

#### Hearing outcome

The 4-PTA (0.5, 1, 2, and 3 kHz) was obtained from the most recent unassisted pure tone audiogram before implantation [5, 27]. The Word Recognition Scores (WRS) of monosyllabic words from both unassisted and aided speech perception audiograms before implantation were also collected at 50 dB, 65 dB, and 80 dB, respectively. The functional gain curve was assessed one month after CI. The speech perception audiograms with CI were assessed 1, 3, 6, and 12 months after surgery to determine the postoperative WRS at 50 dB, 65 dB, and 80 dB. The hearing outcomes were evaluated by comparing the preoperative aided WRS with best-fitted hearing aids with the postoperative WRS using CI at the different time points (1, 3, 6, and 12 months postoperatively). The contralateral ear was muted for the hearing tests. Additionally, the speech perception with CI was compared throughout the different postoperative time points (1, 3, 6, and 12 months postoperatively). Paired testing was performed, so missing data were excluded. This resulted in some audiometric data having fewer than n = 11 items.

#### Functional level scale

The study utilised the FLS, as per definition of the AAO-committee [5], to evaluate the functional levels of the participants. The FLS classifies the functional level of patients with MD using a 6-point scaling system ranging from one (no impairment) to six (severe impairment) [5].

#### Dizziness Handicap Inventory and vertigo-related VAS

The validated German version of the DHI is a 25-question instrument that objectively assesses vertigo-related disability in patients [29–33]. While subdomains of the DHI can be analysed separately in the original English version, this feature is not yet supported in the German version. Therefore, only the total DHI scale was used as validated assessment tool in this study [29]. The DHI categorises vertigo-related impairments into three tiers: minor (0–30 points), moderate (31–60 points), or severe (61–100 points) [33]. Patients’ subjective evaluation of their vertigo-related impairment was also assessed using a 10-point VAS. The VAS scores ranged from zero (absence of vertigo) to ten (highest level of impairment caused by vertigo).

#### Tinnitus Handicap Inventory and tinnitus-related VAS

The validated German version of the THI is a 25-question instrument which categorises tinnitus symptoms as either slight (THI 0–16 points), mild (THI 18–36 points), moderate (THI 38–56 points), severe (THI 58–76 points), or catastrophic (THI 78–100 points) [34, 35]. Patients’ subjective evaluation of the impairment caused by tinnitus was also assessed using a 10-point VAS. The VAS scores ranged from zero (absence of tinnitus) to ten (highest level of impairment caused by tinnitus).

#### Nijmegen Cochlear Implant Questionnaire

The 60-question instrument NCIQ, a validated tool for assessing the hearing-related QoL in patients with CI with a 100 point-score system [12, 36–39], was used in its validated German version [38, 39]. The assessment included the total score and subdomains of basic sound perception, advanced sound perception, speech production, self-esteem, activity, and social interactions. Hinderink et al.’s official scoring key was utilised for evaluation [36, 37].

### Statistical analysis

For the statistical analysis, IBM SPSS Statistics for Windows, Version 29.0 (IBM Corp., Armonk, NY, USA, 2022) was utilised. A statistical consultation took place at the local Institute of Medical Biometry and Informatics, both before data collection and again during data analysis. The data was presented through descriptive statistical measures. The non-parametric, paired Wilcoxon signed-rank test was applied to test with a level of significance of p < 0.05.

## Results

### Demographics

The median age at CI was 59 years. Ten patients with MD with eleven implanted ears (eight males and two females), were included. Seven left ears and four right ears affected by MD were provided with CI. Table 1 displays these data.

**Table 1:** Patient demographics

Age at implantation, gender	Year of CI	Duration of impaired hearing [months]	Side of implantation	Side of MD	Hearing rehabilitation of the contralateral ear	Treatment method for MD prior to CI on the affected ear (1–3 years prior to CI)	Treatment method for MD concomitant with CI	Cochlear implant
48, m	2019	18	Left	Left	–	BH; IC	–	Cochlear CI522
55, m	2018	48	Left	Left	CI	BH; OC; IG	–	Med El Synchrony Flex 28
43, m	2020	48	Right	Right	–	BH; OC; IC	–	Med El Synchrony 2 Flex 28
74, m	2017	84	Right	Bilateral	CI (Implanted contralaterally before 2014)	BH; OC; ESD	–	Med El Synchrony Flex 28
59, m	2015	NA	Left	Left	–	BH	ESD	Med El Synchrony Flex 28
69, f	2016	12	Left	Bilateral	CI (Implanted contralaterally before 2014)	BH; ESD; IG; Surgical neurectomy	–	Cochlear CI522
59; 62, m	2018; 2021	12; 48	Both	Bilateral	CI	BH; OC; IC	–	R: Med El Synchrony Flex 28 L: Med El Synchrony 2 Flex 28
52, m	2015	120	Right	Right	BTE	BH; IG	ESD	Med El Synchrony Flex 28
71, f	2015	> 120	Left	Left	BTE	BH; OC	–	Med El Synchrony Flex 28
52, m	2018	72	Left	Left	–	BH; OC; ESD; IG	–	Med El Synchrony Flex 28
Summary								
n = 11 Age: 59 (43–74; 13.5) 8 × male 2 × female			7 × left 4 × right		4 × CI 2 × BTE 4 × none	10 × BH 6 × OC 4 × IG 3 × IC 3 × ESD 1 × surgical neurectomy	2 × ESD with CI	2 × Cochlear 9 × MedEl

Age at cochlear implantation (CI), gender (male: m; female: f), year of CI, and the duration of impaired hearing in months are displayed. The duration of hearing impairment measures subjective time before CI from when the conventional hearing aid no longer adequately rehabilitated the hearing loss from the patients’ perspective. Additionally, the implanted side affected by Ménière’s disease (MD), and the mode of hearing rehabilitation on the contralateral ear are shown. Treatment methods for MD involved those conducted 1–3 years before CI on the affected ear, and those carried out as one-stage treatment concomitantly with CI. The manufacturers of the CI are listed. The bottom line provides a summary of the data with the median age (minimum–maximum, interquartile range)

*BTE* behind-the-ear hearing aid, *BH* betahistine, *IC* intratympanic corticosteroid injection, *OC* oral corticosteroid intake, *IG* intratympanic gentamicin injection, *ESD* endolymphatic sac decompression

### General

Six patients reported a fast-progressing hearing loss within two years prior to CI, while five patients reported a hearing loss that had slowly progressed over a period of more than two years.While seven patients experienced fluctuating hearing before receiving CI, only three patients experienced this postoperatively. Unilateral MD was present in all of these three patients with postoperative fluctuating hearing. Overall, the cohort reported a high level of satisfaction with the CI. However, five patients noted that their social isolation remained unchanged with the CI and that their hearing in noisy environments did not improve significantly. This data is presented in Table 2.

**Table 2:** Patients’ reports

Question	Preoperatively	Postoperatively
How did the hearing loss evolve?	6 × fast progressing 5 × slowly progressing	–
How stable was your hearing before implantation and how is it now with CI?	7 × fluctuating 4 × stable	3 × fluctuating 8 × stable
Have your speech perception and ability for communication improved after implantation?	11 × yes, improved (100%)	
Do you like to wear your cochlea implant?	–	9 × yes 2 × no (unpleasant because synchronous wearing of glasses and/or face masks)
How good can you deal with everyday life now compared to the time before implantation?	8 × clearly better 2 × slightly better 1 × no change	
Is your cochlea implant indispensable for everyday life?	11 × yes	
How did social isolation change after the implantation?	6 × improved 5 × same level	
How is the hearing with the implant in noisy surroundings compared to the time without implant?	6 × clearly better 5 × no significant change	
Did the directional listening change?	5 × improved clearly 4 × improved slightly 1 × no change 1 × clearly worse	
Do you have pain in the area of your implant?	5 × never 4 × rarely 2 × sometimes	
Are you experiencing any difficulties with your cochlea implant?	1 × difficulties when wearing a hat 1 × no hearing when I’m swimming	
Would you choose the cochlea implantation again for hearing rehabilitation?	11 × yes	

Questions inquiring for patients’ hearing history, subjective satisfaction and experiences with cochlear implant. All ten patients responded to the questions, encompassing a total of eleven ears. Fast progressing hearing loss was defined as hearing impairment occurring within one to 24 months. Slowly progressing hearing loss was defined as hearing loss developing over 2 or more years

### Ménière history

Previous MD treatment history within the cohort is shown in Table 1. All ten patients had taken betahistine 1–3 years prior to CI. Six patients had taken oral corticosteroids, four had received intratympanic gentamycin. Three patients had an intratympanic corticosteroid injection, three had endolymphatic sac decompression and one had a surgical neurectomy elsewhere prior to CI. Two patients underwent endolymphatic sac decompression simultaneously with CI. Regarding the individual most disabling MD symptoms eight patients reported hearing loss as most disabling, eight reported vertigo symptoms as most disabling, seven reported nauseas during attacks, and six reported many days off to work and social life as most disabling (n = 11).

### Hearing outcome

#### Pure tone audiometry

The median unaided preoperative 4-PTA measured 91.5 dB (Table 3), indicating an overall MD stage of four as per the AAO-HNS Equilibrium Committee [5].

**Table 3:** Pre- and postoperative hearing data

		Median (min, max, IQR) [%]	Mean ± SD (SE) [%]	95% Confidence interval (lower bound; upper bound) [%]	n
	Preop data				
	4-PTA unaided [dB] (0.5, 1, 2, 3 kHz)	91.5 (60.250;130.0;45.250)	94.2 ± 25.10 (7.937)	76.542; 111.913	11
	WRS preop [%]				
Unaided	50 dB	0 (0; 0; 0)	0 ± 0 (0)	0; 0	10
	65 dB	0 (0; 0; 0)	0 ± 0 (0)	0; 0	10
	80 dB	0 (0; 0; 0)	0 ± 0 (0)	0; 0	10
Aided	50 dB	0 (0; 5; 0)	0.63 ± 1.768 (0.625)	– 0.85; 2.1	8
	65 dB	0 (0; 35; 0)	4.38 ± 12.374 (4.375)	– 5.97; 14.72	8
	80 dB	2.5 (0; 55; 48)	17.5 ± 24.64 (8.712)	– 3.1; 38.1	8
	Postop data with CI				
	Functional gain curve (0.5;1;2;3 kHz)	44 (31.250; 85.0; 11.0)	46.864 ± 14.185 (4.486)	36.573; 56.654	11
	WRS postop [%]				
1 mo postop	50 dB	0 (0; 10; 10)	2.86 ± 4.880 (1.844)	– 1.66; 7.37	7
	65 dB	55 (45; 80; 15)	56.43 ± 12.150 (4.592)	45.19; 67.67	7
	80 dB	75 (60; 85; 20)	72.14 ± 10.351 (3.912)	62.57; 81.72	7
3 mo postop	50 dB	0 (0; 15; 7.5)	3.5 ± 5.5 (1.833)	– 0.647; 7.647	10
	65 dB	47.5 (0; 80; 35)	42.5 ± 25.125 (8.375)	23.555; 61.445	10
	80 dB	72.5 (10; 90; 21.25)	64 ± 25.080 (8.360)	45.088; 82.912	10
6 mo postop	50 dB	0 (0; 10; 0)	1.5 ± 3.202 (1.067)	– 0.914; 3.914	10
	65 dB	52.5 (0; 75; 23.750)	45.5 ± 25.244 (8.415)	26.465; 64.535	10
	80 dB	80 (40; 100; 25)	76.5 ± 20.622 (6.874)	60.950; 92.050	10
12 mo postop	50 dB	0 (0; 45; 2.5)	5.909 ± 13.111 (4.146)	– 3.329; 15.147	11
	65 dB	55 (25; 95; 62.5)	53.182 ± 19.22 (6.078)	39.639; 66.724	11
	80 dB	75 (40; 95; 25)	75.909 ± 17.428 (5.511)	63.630; 88.189	11

The data presented here includes preoperative (preop) hearing outcomes as unaided 4-pure tone audiometry (4-PTA), and unaided and aided (with best-fitted hearing aid) word recognition scores (WRS) of monosyllabic words in percent [%] at 50 dB, 65 dB, and 80 dB, respectively

Additionally, postoperative (postop) hearing outcomes are displayed: functional gain curve with cochlear implant (CI), and the WRS of monosyllabic words in percent [%] 1, 3, 6, and 12 months (mo) postoperatively at 50 dB, 65 dB, and 80 dB, respectively. The data is expressed as median, minimum (min), maximum (max), interquartile range (IQR), mean ± standard deviation (SD), and standard error (SE). 95% confidence intervals with upper and lower bounds are shown

#### Functional gain curve

The median functional gain curve one month after CI was 44 dB (Table 3).

#### Word recognition scores

**Fig. 2:**
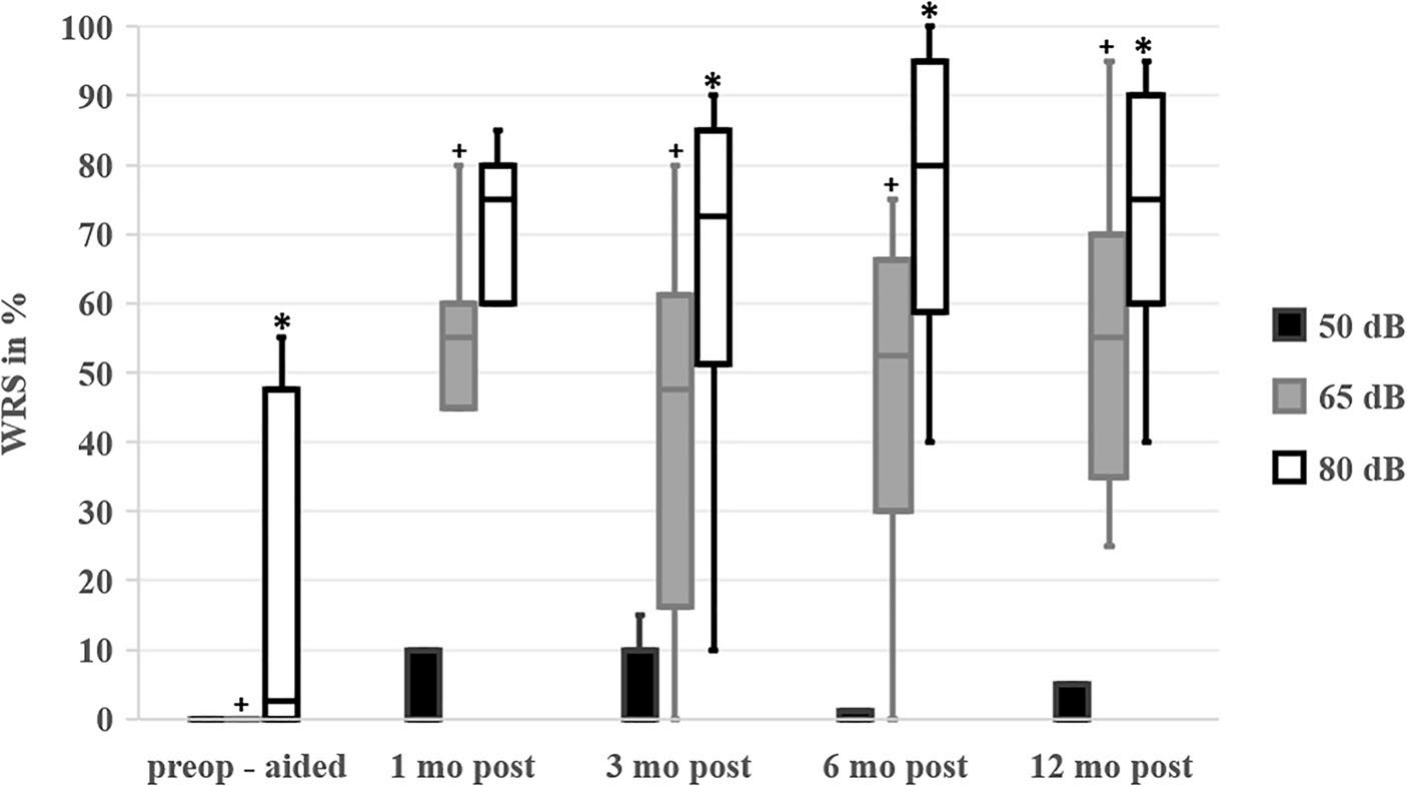
Word Recognition Scores (WRS). WRS of monosyllabic words are displayed in percent [%] at sound pressure levels of 50 dB (black boxes), 65 dB (grey boxes), and 80 dB (white boxes). The WRS were measured before implantation with the best-fitted hearing aid (preop-aided), and at 1, 3, 6, and 12 months (mo) after implantation (postop) with the cochlear implant. The plus signs + indicate statistical significance (p < 0.05) in WRS at sound level pressures at 65dB when comparing preoperative and postoperative WRS at different time points. The stars * indicate statistical significance (p < 0.05) in WRS at sound level pressures at 80 dB when comparing preoperative and postoperative WRS at different time points. There was no statistical significance between the pre- and postoperative comparisons at 50 dB. There was no statistical significance within the postoperative WRS at different time points. The p-values can be found in Table 4

A comparison between the preoperative WRS with best-fitted hearing aids (Table 4) and the postoperative WRS with cochlear implant 1, 3, 6, and 12 months after implantation depicts a significant enhancement of speech perception at 65 dB and 80 dB HL which was not observed at sound levels of 50 dB (Table 4, Fig. 2). The comparison of the preoperative aided WRS at 80 dB to the WRS 1 month postoperatively did not show statistical significance (Table 4, Fig. 2). There was a trend, although not statistically significant, towards even better speech perception within the first year of CI (Fig. 2).

**Table 4:** P-Values of testing the hearing outcomes

	Sound pressure level			n
	50 dB	65 dB	80 dB	
p-values				
Preop vs. 1 month postop	0.655	**0.042**	0.063	5
Preop vs. 3 months postop	0.180	**0.016**	**0.016**	7
Preop vs. 6 months postop	1.00	**0.027**	**0.031**	7
Preop vs. 12 months postop	0.180	**0.012**	**0.012**	8
1 month postop vs. 3 months postop	1.000	1.000	0.197	7
1 month postop vs. 6 months postop	0.785	0.684	0.292	6
1 month postop vs. 12 months postop	0.465	0.670	0.288	7
3 months postop vs. 6 months postop	0.336	0.553	0.092	9
3 months postop vs. 12 months postop	0.588	0.151	0.123	10
6 months postop vs. 12 months postop	0.180	0.374	0.779	10

Comparison of hearing outcomes before (preop) and after (postop) cochlear implantation (CI) using the non-parametric, paired Wilcoxon signed-rank test

Significant changes (p < 0.05) are highlighted in bold labels. No Bonferroni correction was implemented, as advised by the consulting statistician from the local Institute of Medical Biometry and Informatics due to the small sample size

### Functional level scale

The FLS [5] improved significantly (p = 0.020) postoperatively from a median of five to a median of three (n = 8).

### Dizziness Handicap Inventory and vertigo-related VAS

The DHI scores improved significantly (p = 0.043) from severe (median: 65 points) preoperatively to moderate (median: 37 points) postoperatively (n = 8). Similarly, there was a significant improvement in the subjective VAS score for vertigo impairment, from a median of nine preoperatively to a median of three postoperatively (p = 0.017).

### Tinnitus Handicap Inventory and tinnitus-related VAS

The THI scores improved significantly (p = 0.043) from moderate (median: 47 points) preoperatively to mild (median: 27 points) postoperatively (n = 8). Based on the VAS, patients reported an improvement in their tinnitus impairment following implantation, with the median score decreasing from eight preoperatively to six postoperatively (p = 0.042).

### Nijmegen Cochlear Implant Questionnaire

**Fig. 3:**
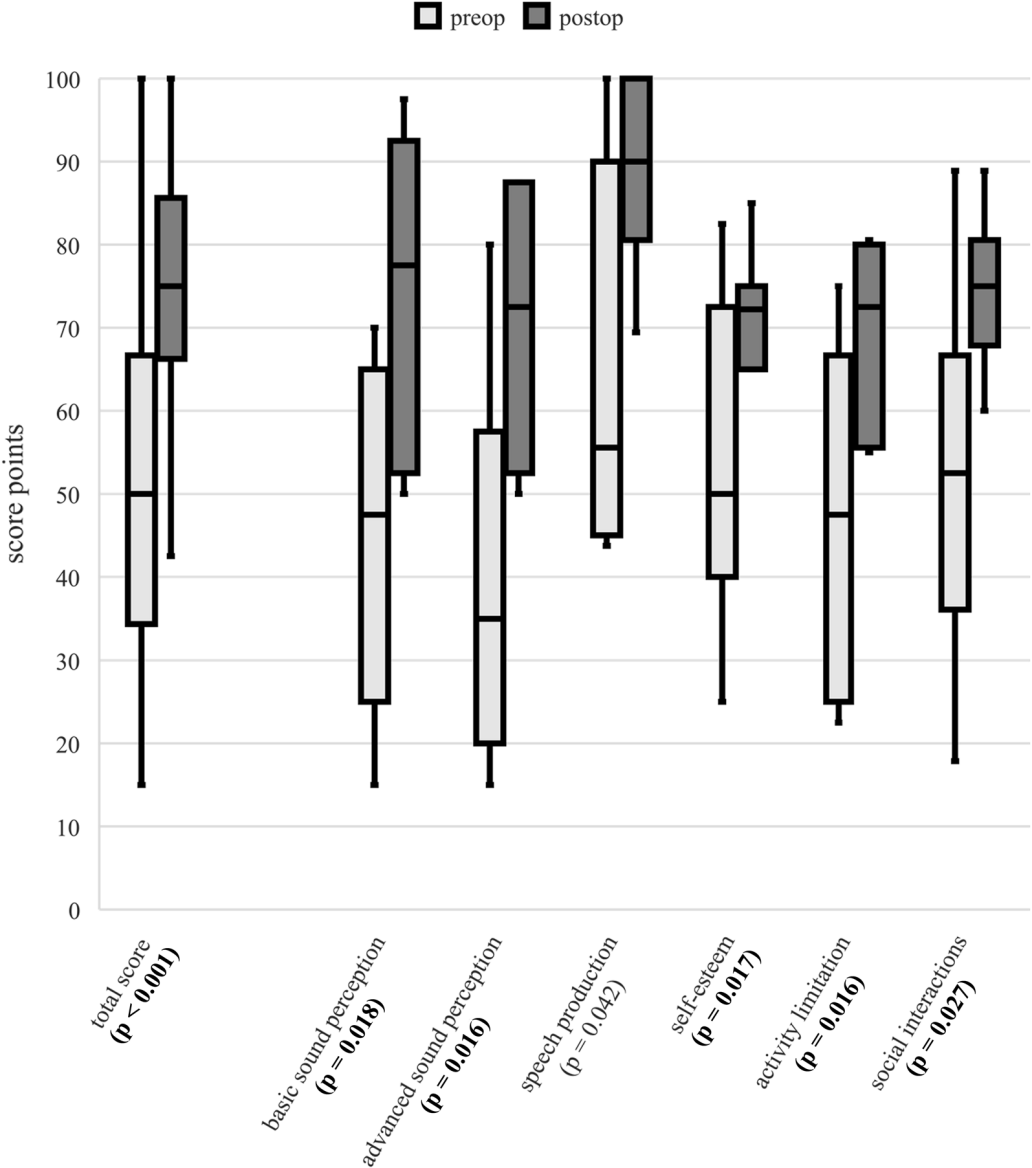
Pre- and postoperative results of the Nijmegen Cochlear Implant Questionnaire. The postoperative total score and all postoperative subdomain scores with score points from zero to 100 on the y-axis [score points] improved significantly compared to the preoperative scores at significance level of p < 0.05. The p-values comparing the pre- and postoperative values are given in brackets after each of the x-axis labels

The NCIQ showed a significant improvement in hearing-related QoL in all subdomains as well as in the total score (Fig. 3). The total score improved from 50.0 points in median preoperatively to 75.0 points postoperatively (p < 0.001). The basic sound perception improved from 47.5 points in median preoperatively to 77.5 points in median postoperatively (p = 0.018), the advanced sound perception from 35.0 points to 72.5 points (p = 0.016), the speech production from 55.6 points to 90.0 points (p = 0.042), the self-esteem from 50.0 points to 72.2 points (p = 0.017), the activity limitation from 47.5 points to 72.5 points (p = 0.016) and social interactions from 52.5 points to 75.0 points (p = 0.027). Three patients had to be excluded due to incomplete responses on the NCIQ (n = 7).

## Discussion

A large number of high-quality research articles have been published on the topic of MD and CI. As MD does not only lead to hearing impairment, but also to impairment in other aspects, it remains important to focus not only on the hearing outcome in rehabilitation programmes for MD patients, but also to focus on the concomitant problems that patients with MD face, namely vertigo and tinnitus. It is already known that patients with MD benefit from their CI not only in terms of hearing, but also in terms of QoL, tinnitus and vertigo [16, 18]. Most studies on CI in patients with MD have so far focused on hearing outcomes or on other singular outcome aspects such as tinnitus, vertigo or functional levels and, surprisingly, have often used unvalidated instruments for data collection. Fife et al. assessed the FLS and the Hearing Handicap Inventory, but did not perform a validated outcome assessment of vertigo and tinnitus [21]. Similarly, Kurz et al. examined the FLS and the QoL with the Morbus Ménière Outcome questionnaire, but did not use validated tools for reporting the outcomes of vertigo and tinnitus [15]. McRackan et al. and Lustig et al. investigated the effects of vertigo and tinnitus in relation to patient-reported symptom changes after CI [6, 14]. However, they did not utilize validated tools, such as the DHI or the THI [6, 14]. Canzi et al. and Mick et al. used the THI for validated evaluation of tinnitus outcome in MD patients with CI [17, 26]. Canzi et al. and Doobe et al. used the DHI for validated assessment of vertigo in MD patients with CI [26, 28]. According to Villavisanis et al.’s review of 37 studies on CI in MD, only two used the THI [17, 26] and one used the DHI [26] as validated assessment tools for vertigo and tinnitus [23]. The NCIQ has been a precise tool for evaluating hearing-related QoL in patients with CI [36–39] in the previous decade. However, the NCIQ has rarely been used to report the QoL related to hearing of patients with MD who have received CI [16]. Desiato et al.’s review found that only one out of four studies utilized the NCIQ for assessing the QoL related to hearing in MD patients with CI [16]. In order to gain a comprehensive insight into all possible areas of impairment in patients with MD, we examined the outcome of our cohort using validated outcome tools for vertigo, tinnitus and hearing-related QoL in addition to hearing outcome measures, and together with the assessment of the subjective vertigo- and tinnitus-related VAS, which to our knowledge is new.

### General

We set the follow-up period to a minimum of twelve months between study entry and cochlear implantation. We felt this was feasible, as Mick et al. also used a twelve-month period for outcome evaluation [17], and others even had only 6 months of postoperative care before enrolment [14, 40].

The patients with MD in our cohort were mainly implanted in the second half of life. The high number of different treatments that patients had undergone in their medical history is indicative of the high burden associated with their MD. The general information received by the patients demonstrated a high level of satisfaction within our cohort, with all the patients indicating that the CI was necessary for their daily lives and they would choose to receive it again. The most severe subjective symptoms experienced by the patients in our cohort were hearing loss and vertigo. Our assessment of the FLS demonstrated a noteworthy enhancement in the overall functioning of MD patients following CI. Since MD patients often have absences from work, causing high direct and indirect costs for the economy, not only the patients themselves profit from improved functional levels, but the whole society [41].

### Hearing outcome

While some patients achieved a good WRS early after CI, others take much longer to achieve a satisfactory WRS. Considering that the duration of deafness plays an important role in postoperative speech perception with CI [12], the WRS achieved in this study may be dependent on the individual hearing history prior to implantation. However, the amount of data available in this study is small (n = 11), which is a limitation of the results presented.

The evaluation of the preoperative 4-PTA revealed that the implanted patients in our cohort all had advanced MD, with a minimum of stage three and a maximum of stage four according to the classification defined by the AAO-HNS Equilibrium Committee [5]. A comparison of the preoperative median 4-PTA (91.5 dB) and the postoperative median functional gain curve (44 dB) showed a clear improvement in basic sound perception.

Speech comprehension with CI was significantly better after implantation compared to preoperative aided speech comprehension. Although speech comprehension remained difficult for our patients with MD and CI at low sound pressure levels of 50 dB, significant improvements in WRS were observed at levels of 65 dB and 80 dB.

Within the first year, speech perception improved slightly further, which could be attributed to the positive training effect over time.

Our study confirms that MD patients with CI have a clear hearing benefit, as has been reported previously [6, 14, 19, 20]. However, other areas of outcome measures have also improved, as discussed below.

### Outcome of vertigo, tinnitus and hearing-related quality of life

#### The outcome of vertigo

As previously observed by other researchers [14–17], our cohort demonstrated a reduction in vertigo symptoms from “severe” to “moderate” following implantation, indicating a positive effect of CI on the vestibular symptoms of patients [29–32]. Both the subjective VAS and the validated DHI indicated a postoperative improvement in vertigo in our cohort. At our department, we did not perform simultaneous surgical labyrinthectomy with CI due to the destructive nature of such a procedure [22]. However, studies have reported improvements in vestibular symptoms in cases where simultaneous labyrinthectomy was performed [18, 22]. It remains challenging to determine precisely how CI affects the vestibular organ and vertigo [15–17]. Nonetheless, patients with MD who undergo CI benefit from their CI, encountering not just elevated hearing results but also improved vestibular symptoms. Improved directional hearing after hearing rehabilitation through cochlear implantation may also play a role in patients reporting better spatial orientation or less vertigo, respectively, after CI.

#### The outcome of tinnitus

Due to the subjective nature of tinnitus symptoms, it is challenging to assess tinnitus-related impairments in affected patients [34, 35]. Validated questionnaires aid in categorising tinnitus symptoms of patients into different degrees of severity [34, 35]. After CI in patients with MD, an improvement in tinnitus symptoms was reported [16, 17]. This was also demonstrated in our cohort, where the tinnitus-related impairment decreased from "moderate" to "mild" [35, 42–44]. Both the subjective VAS and the validated THI indicated an improvement in tinnitus postoperatively in our cohort. The reasons for the improvement in tinnitus after CI remains unclear. One possibility could be a reduction of auto stimulation in the auditory pathway, as CI reintroduces meaningful input.

#### The outcome of hearing-related quality of life

CI in general [12, 39] and CI in MD patients are known to improve hearing-related QoL [16, 17], regardless of the duration of deafness [12]. As some patients with MD still suffer from other Ménière's symptoms after implantation, such as a persistent vertigo, the improvement in hearing-related QoL in CI patients with MD has been reported to be less compared to CI patients without MD [17]. In our cohort, the total score and all subdomains of the NCIQ improved significantly, in agreement with previous studies [12, 36, 37]. When comparing pre- and postoperative levels in the subdomains, Plath et al. reported the lowest difference in the "advanced sound perception" domain in their cohort of patients with CI, without specifying the presence of MD [39]. In our cohort, however, the lowest difference between pre- and postoperative scores was found in the “social interaction” subdomain. The reason for this may be that patients with a long history of MD tend to be inactive people due to the social withdrawal they have suffered from their disease for years. This condition may not be changed by CI, as isolation due to social withdrawal is not easily reversed. In this cohort we found the highest difference of 375 points in the pre-post comparison of the subdomain "advanced sound perception". This is in line with Hinderink et al. [37] and Hirschfelder et al. [12]. The reason for this may be that hearing in challenging environments clearly improved after CI as binaural hearing may not have been possible prior to implantation.

### Limitations

This study has several limitations. As our study only included patients with MD who received implants, there may be a potential for selection bias. It was very difficult to recruit patients who met the strict inclusion criteria of having a definite MD and having received a CI at our centre between 2014 and 2022. Patient recruitment was also made more difficult by the coronavirus pandemic. Only ten patients and eleven ears could be included, which is a rather low number. The low number of MD patients with CI in studies is a problem already known from previous studies [6, 16, 17]. Kurz et al. reported on eight patients [15], Fife et al. had a cohort of ten patients [21], Lustig et al. included nine patients of whom seven were bilaterally affected [6], Hansen et al. had ten patients [22], Mick et al. studied 20 patients [17] and McRackan et al. 21 patients [14]. Villavisanis et al. reviewed 37 studies and could only include 216 MD patients with CI [23]. Desiato et al. reviewed 17 studies on MD patients with CI and could only include a total of 182 patients [16], resulting in approximately ten to eleven patients per study. This is consistent with the number of MD patients in our cohort who underwent CI. This shows that enrolment of MD patients with CI is a general problem, as the diagnosis of definite MD itself is not common and not every MD patient receives a CI.

As the patient cohort was small, some of the observations in this study may not reflect the results that a whole population of patients with MD after CI would show. For example, the mean WRS at 3 months postoperatively at 65 dB and 80 dB was lower than the scores at 1 month and 6 months postoperatively, despite one would expect a gradual improvement of WRS as patients acclimatize to their CI over time. Here, the limited cohort size is once again a constraint.

Another limitation is the retrospective and uncontrolled nature of this study. There is a likely phenomenon of response shift and recall bias in these data [40, 45]. Brill et al. demonstrated the presence of the response shift phenomenon in CI users [40]. They found that CI users may retrospectively overestimate their hearing-related QoL with CI, as measured by the NCIQ, particularly in the psychological and social domains [40]. But they also reported that the tested responses had the same direction as the actual observations [40]. Therefore, despite the retrospective nature of the data, retrospective evaluations of the hearing-related QoL can estimate the correct tendency of the development of patients’ QoL under CI. We conclude, despite the potential presence of response shift effects, that the results of this study can reliably reflect the actual improvements in hearing-related QoL in our small cohort.

The subjective nature of the VAS and the DHI, THI and NCIQ is a further limitation. This is a general problem with VAS-/questionnaire-based data. Some patients may not have been able to provide a realistic assessment of their impairment [39], either because some of the patients who underwent cochlear implantation in earlier years may not have a good recollection of how their hearing and symptoms of tinnitus and vertigo were before implantation, or because they have had MD for such a long time that they could not compare it to a time when they were asymptomatic. However, the assessment of VAS scores for vertigo and tinnitus, as well as the DHI and THI scores, showed a positive trend towards less vertigo- and tinnitus-related disability postoperatively. This suggests an improvement in these aspects.

Patients reported postoperative improvement in their vertigo. However, objective vestibular function tests were not regularly performed postoperatively in our patient cohort, so we cannot report any objective changes in postoperative vestibular function. Including these tests in future studies could improve the evaluation of postoperative vertigo in patients with MD who have undergone CI. It is known that CI can impact vestibular function [25, 46] and aggravate existing MD [25]. Given this background and the fact that our study showed an improvement in vertigo-related impairment based on subjective vertigo assessment, it would be interesting to compare the subjective and objective vertigo assessments in future studies.

The strengths of the study were the comprehensive and detailed description of a wide range of outcome measures using mainly validated instruments.

## Conclusion

MD patients benefit from CI not only in terms of hearing outcome but also in terms of associated symptoms such as vertigo and tinnitus. Discrimination of speech at low sound levels remained difficult for MD patients with CI, whereas good results were obtained at speech levels of 65 dB and 80 dB. Hearing loss and vertigo were the main disabling symptoms for the MD patients in our cohort, and both hearing and vestibular symptoms improved postoperatively. CI in our MD patients led to better functional levels and thus better participation in life and society.

Due to the retrospective nature and small number of patients, not only in this study but also in other comparable studies, multicentre studies with a prospective study design should be carried out in the future. The use of validated instruments to assess vertigo, tinnitus and hearing-related QoL in MD patients with CI should be standardised in the future and firmly integrated into the everyday clinical practice.

## Data Availability

Not applicable.
