# Correction: Long-term outcome of patients with Ménière’s disease following cochlear implantation: a comprehensive outcome study with validated assessment tools

**DOI:** 10.1007/s00405-024-08814-7

**Published:** 2024-08-13

**Authors:** Miray-Su Yılmaz Topçuoğlu, Peter K. Plinkert, Mark Praetorius, Sara Euteneuer

**Affiliations:** 1 Department of Otorhinolaryngology, University Hospital Heidelberg, Im Neuenheimer Feld 400, 69120 Heidelberg, Germany; 2 Department of Otorhinolaryngology, University Hospital Hamburg-Eppendorf, Hamburg, Germany

**Correction: European Archives of Oto-Rhino-Laryngology** 10.1007/s00405-024-08690-1

In this article Fig. 2 was incorrectly processed during the production process; the Fig. 2 should have appeared as shown below.

**Fig. 2:**
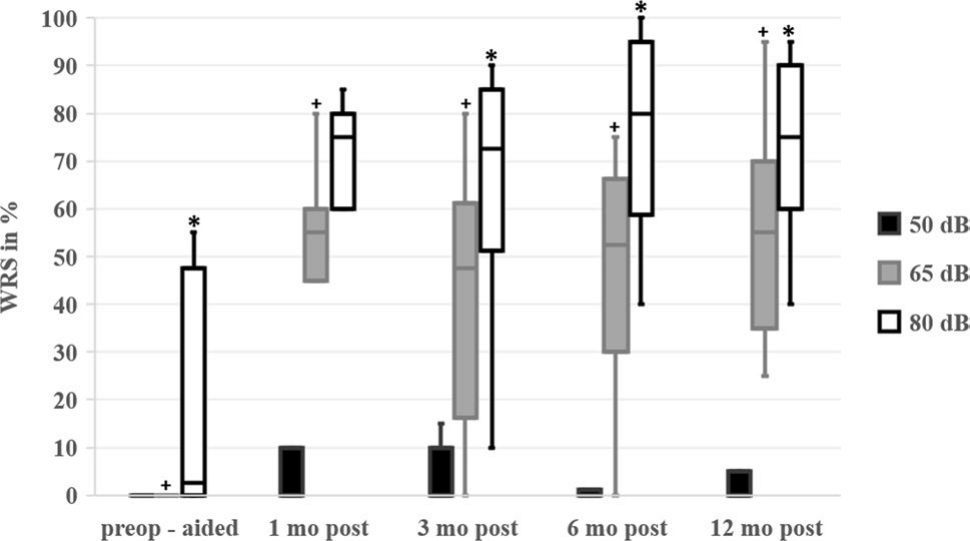
Word Recognition Scores (WRS). WRS of monosyllabic words are displayed in percent [%] at sound pressure levels of 50 dB (black boxes), 65 dB (grey boxes), and 80 dB (white boxes). The WRS were measured before implantation with the best-fitted hearing aid (preop-aided), and at 1, 3, 6, and 12 months (mo) after implantation (postop) with the cochlear implant. The plus signs + indicate statistical significance (p < 0.05) in WRS at sound level pressures at 65 dB when comparing preoperative and postoperative WRS at different time points. The stars * indicate statistical significance (p < 0.05) in WRS at sound level pressures at 80 dB when comparing preoperative and postoperative WRS at different time points. There was no statistical significance between the pre- and postoperative comparisons at 50 dB. There was no statistical significance within the postoperative WRS at different time points. The p-values can be found in Table 4

The original article has been corrected.

